# A rare case of giant pelvic retroperitoneal schwannoma

**DOI:** 10.1016/j.radcr.2024.08.109

**Published:** 2024-09-11

**Authors:** Takashi Kawahori, Shoichiro Mukai, Yasufumi Saito, Toshihiro Nishida, Toshikatsu Fukuda, Hideki Ohdan

**Affiliations:** a Department of Surgery, Chugokurosai Hospital, 1-5-1, Hirotagaya, Kure City, Hiroshima 737-0193, Japan; b Department of Pathology, Chugokurosai Hospital, 1-5-1, Hirotagaya, Kure City, Hiroshima 737-0193, Japan; c Department of Gastroenterological and Transplant Surgery, Graduate School of Biomedical and Health Sciences, Hiroshima University, 1-2-3 Kasumi, Minami-ku, Hiroshima 734-8551, Japan

**Keywords:** Giant schwannoma, Retro-peritoneal tumor, Embolization

## Abstract

This report highlights the successful treatment of large pelvic schwannomas and underscores the importance of preoperative embolization. A 40-year-old male presented with a lower abdominal mass and reported pain and numbness in the left lower limb attributed to nerve compression. Preoperative embolization of the main feeding vessels was performed to mitigate intraoperative bleeding. The tumor was completely excised during surgery, with preservation of the hypogastric nerve. Histopathological examination confirmed the diagnosis of schwannoma. We underscore the significant role of preoperative embolization in effectively managing large pelvic tumors, such as schwannomas, by reducing the risk of excessive bleeding.

## Introduction

Schwannomas rarely but occasionally arise among pelvic tumors, primarily in the retroperitoneal space [1,2]. While most schwannomas are benign, a definitive diagnosis requires a histopathological examination. Careful consideration is essential during tumor resection, as the nerve of origin may lead to serious complications.

Large pelvic tumors are anatomically proximate to other organs and often feature abundant feeding vessels, necessitating preparation for potential intraoperative injury to the adjacent organs and significant bleeding. We herein report a case in which the tumor was completely resected using interventional radiology after preoperative embolization of the main feeding vessels.

The patient experienced a favorable outcome, with no neurological damage. This approach highlights the importance of strategic planning and collaboration between surgical and interventional radiology teams for managing complex pelvic schwannomas.

## Case presentation

**Fig. 1:**
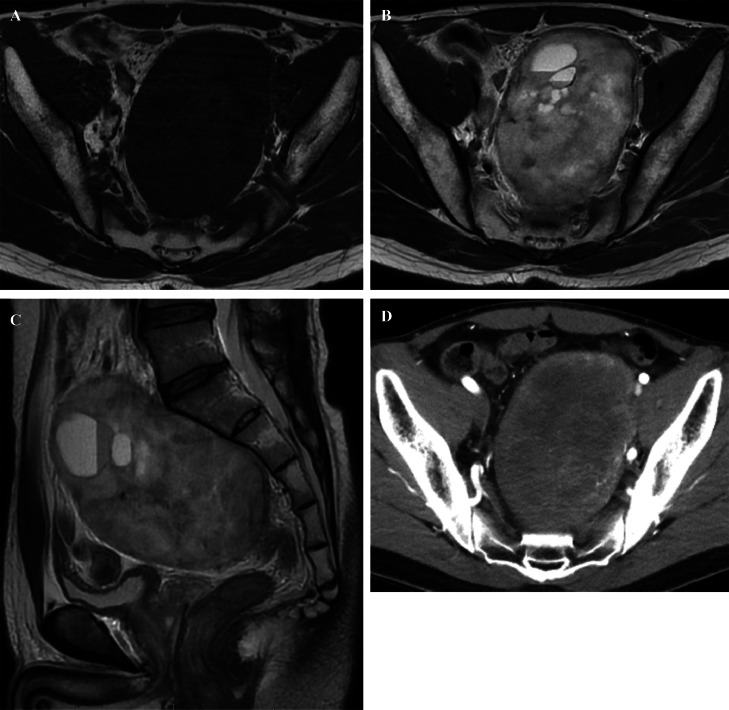
(A) MRI T1-weighted axial image showed a tumor with a well-defined marginal membrane and low signal inside the tumor. (B) MRI T2-weighted axial image showed mainly heterogeneous high signal inside the tumor, with a mixture of substantial areas and cysts. (C) MRI T2-weighted sagittal image showed the tumor in contact with the first sacrum. (D) CT showed a tumor with a diameter of 11.5 × 8 cm occupying the pelvic cavity.

**Fig. 2:**
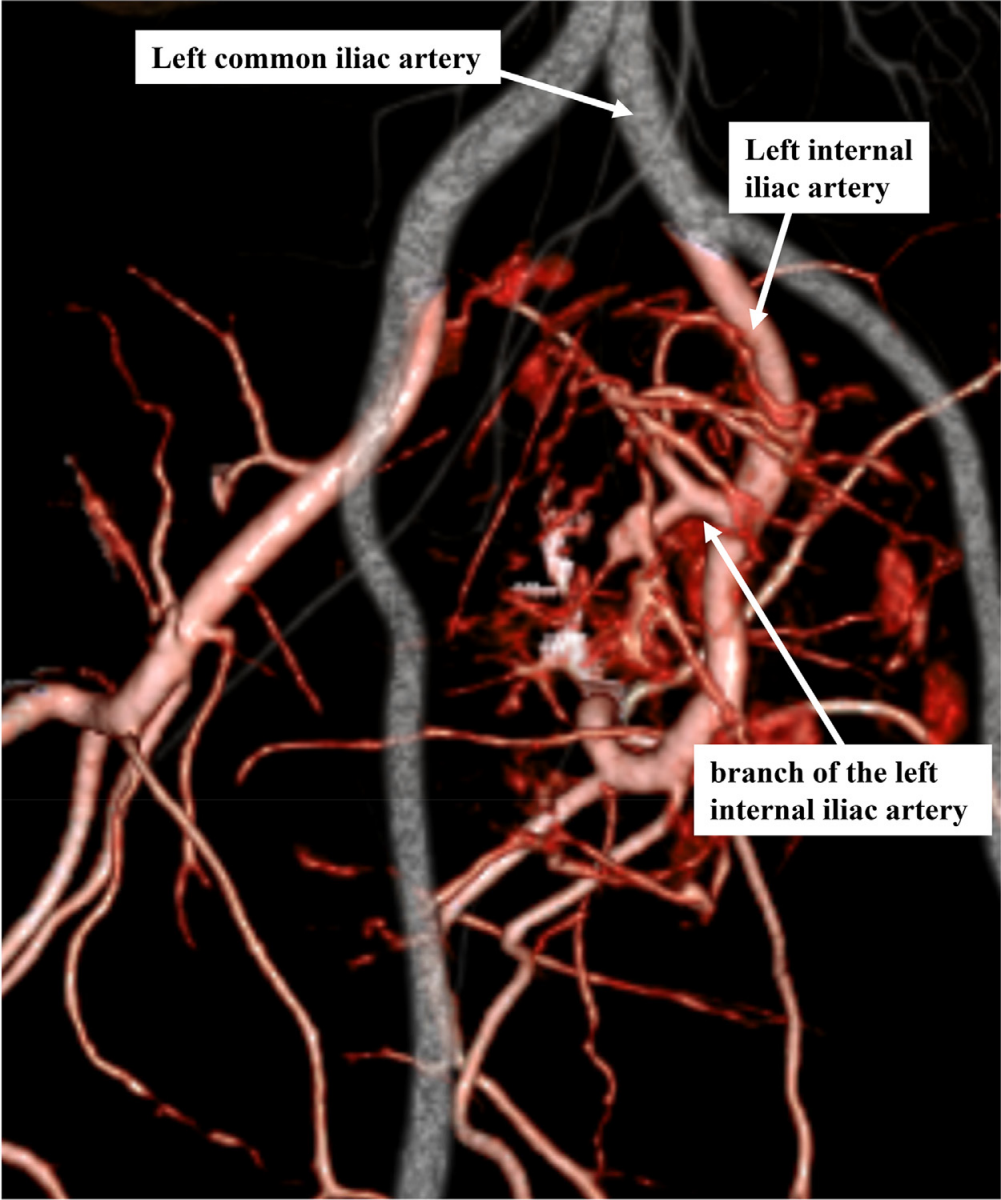
Contrast-enhanced CT 3D vascular reconstruction image of the common iliac artery region. The tumor was highly perfused, and the main feeding artery was a branch of the left internal iliac artery.

**Fig. 3:**
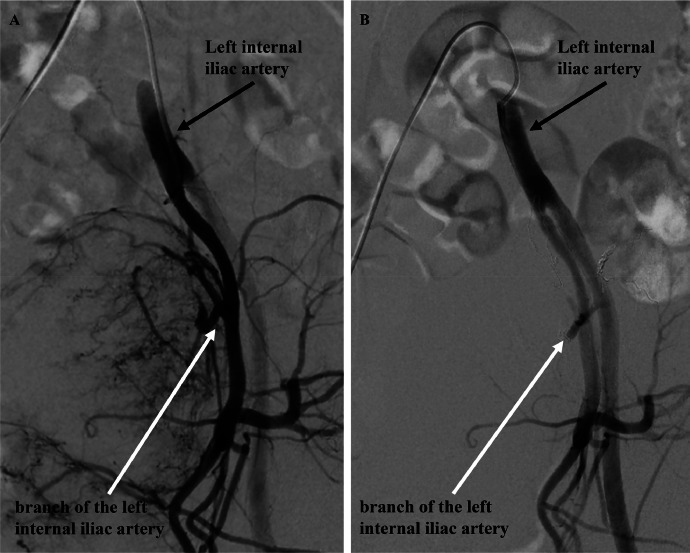
(A) Angiographic image from the left internal iliac artery. The tumor had abundant blood flow from the left internal iliac artery. (B) Postembolization image of a branch of the left internal iliac artery. Blood flow into the tumor was almost completely eliminated by embolization.

A 40-year-old male, with an unremarkable medical history, was referred to our hospital for a thorough examination following an ultrasound scan during his medical checkup, which revealed a mass in his lower abdomen. Magnetic resonance imaging (MRI) disclosed a tumor characterized by clear borders, well-defined margins, and a maximum diameter of 13 cm. T2-weighted images of the tumor's interior exhibited heterogeneous high-signal content with a mix of substantial areas and cysts. The margins displayed a capsular structure with a low signal on both T1-weighted and T2-weighted images, consistent with a schwannoma (Figs. 1A-C). Computed tomography (CT) illustrated a large tumor measuring 11.5 × 8 centimeters (cm), occupying the pelvic region (Fig. 1D). The ureter and rectum were situated ventral to the tumor, indicating a primary retroperitoneal origin. No evident invasion or distant metastasis to other organs was observed. The feeding vessel of the tumor was identified as a branch of the left internal iliac artery (Fig. 2). During the waiting period for surgery, the patient developed pain and numbness in the left lower limb, symptoms attributed to nerve compression caused by the tumor. Due to the tumor's size, significant blood flow, and the anticipation of massive intraoperative bleeding, embolization was performed by interventional radiology (IVR) 3 days before surgery (Figs. 3A and B). Angiography was performed to visualize the abdominal aorta, left internal iliac, lumbar, and lateral sacral arteries. Angiography of the left internal iliac artery revealed that the main feeding vessel of the tumor branched off from the left internal iliac artery. When multiple vessels branched from this artery, coil embolization was performed for these vessels. Embolization was performed using a gelatin sponge for the main trunk of the feeding vessel. Subsequent angiography confirmed the absence of tumor enhancement after embolization.

The intraoperative findings, revealed through a laparotomy with a median incision in the lower abdomen, unveiled a sizable tumor exerting pressure on the rectum and ureter while occupying the pelvic cavity. Notably, no disseminated nodules were observed in the abdominal cavity. During rectal dissection, the outer capsule of the tumor ruptured, allowing ingress into the retroperitoneal cavity. The dissection commenced ventrally, focusing on removing the outer capsule from the tumor's surface. The upper edge of the tumor was situated proximal to the bifurcation of the left internal and external iliac arteries. Predominantly affixed to the pelvic wall on the dorsal and left sides, the tumor was excised meticulously, preserving the capsule integrity. Detachment of the dorsal tumor side involved stripping the sacral periosteum, while removal of the left side required dissection of the connective tissue adjacent to the left internal iliac vein. The entire procedure lasted 168 minutes, with a recorded blood loss of 1130 mL.

**Fig. 4:**
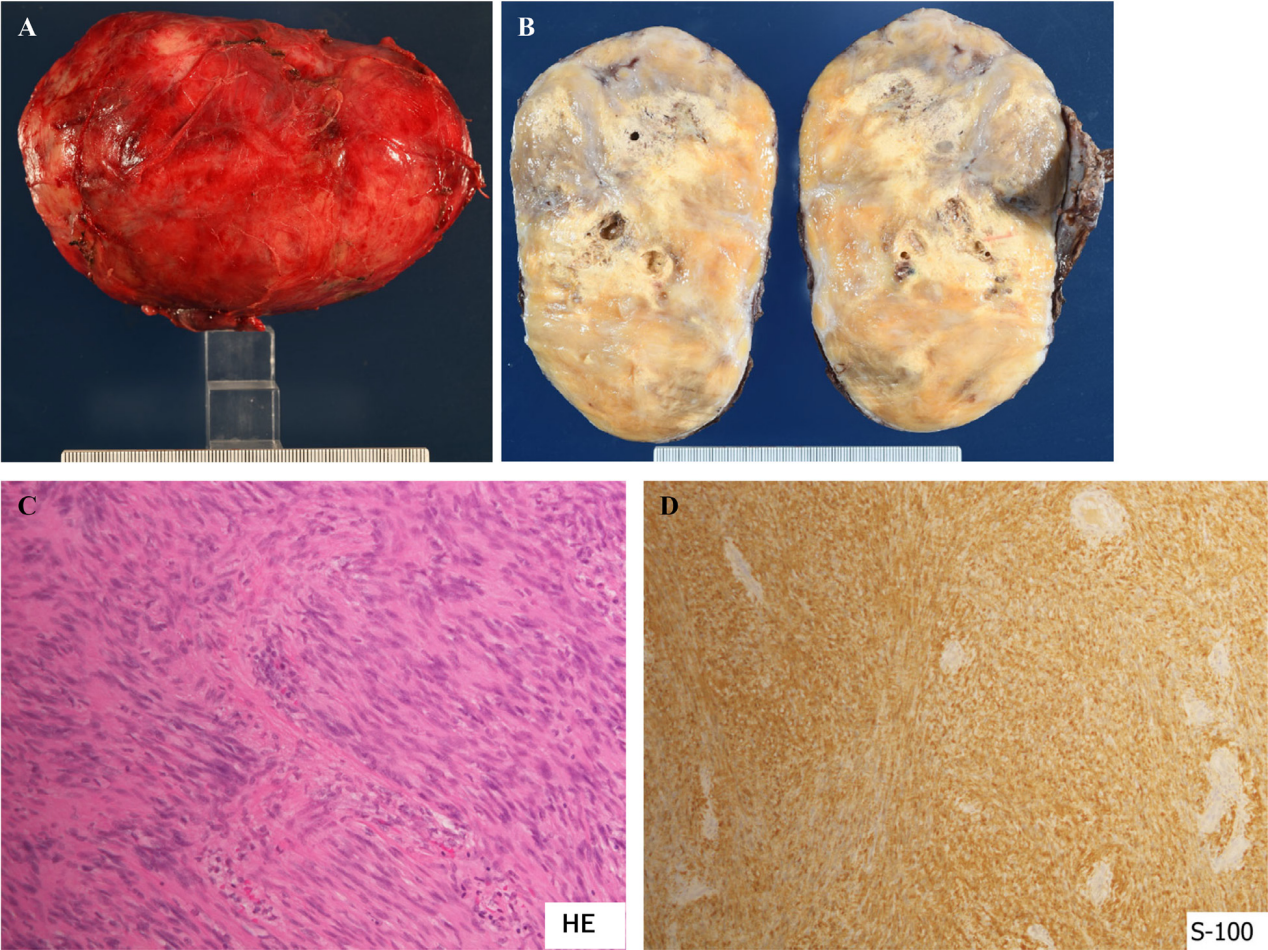
(A) Gross impression of the tumor. The tumor was 13 × 8 cm in size and weighed 473 g. (B) Split view of the tumor. The tumor was a dense tumor covered with a membrane, with some necrosis of the inside. (C) HE image of the tumor. The tumor consisted of a mixture of spindle-shaped cells in a bundle arrangement (Antoni A) with a poorly atypical nucleus (Antoni A) and Antoni B with a poor cellular component, with little or no mitotic features. (D) S-100 immunostaining image. The tumor cells were S-100 positive.

The pathological examination unveiled a well-demarcated tumor encased in a capsule exhibiting partial necrosis (Figs. 4A and B). The tumor manifested a bundle-like arrangement of spindle-shaped cells characterized by indistinct round nuclei (Antoni A) and a mixture of cells with limited cellular components (Antoni B), featuring minimal or absent nuclear mitosis. Immunohistochemistry confirmed S100 positivity in the tumor cells, leading to the diagnosis of schwannoma (Figs. 4C and D). Following surgery, the patient experienced an amelioration of neurological symptoms in the left lower limb, leading to discharge from the hospital on the eighth day without any notable complications.

## Discussion

Schwannomas are benign tumors enveloped by a membrane composed of Schwann cells originating from peripheral nerves and represent the most prevalent tumors of peripheral nerve origin [1,2]. Malignant transformation rarely occurs, except in cases associated with neurofibromatosis type I and melanotic schwannomas [3,4]. Schwannomas can manifest in all age groups, peaking in incidence between the ages of 20 and 50, with no discernible sex or racial predilection [1]. Predominantly located in the head and neck region as well as the extremities, approximately 3%-5% of schwannomas occur in the retroperitoneum [5]. While most patients remain asymptomatic, pressure on adjacent nerves may result in sensory abnormalities, neuropathic pain, and movement disorders [6]. Diagnostic imaging is commonly performed using CT and MRI. CT scans typically reveal a cyst with a smooth surface and contrast effect, exhibiting lower CT values relative to muscle. Hemorrhage and necrosis are frequently observed within the tumors. In contrast, MRI T1-weighted images display an intermediate signal intensity akin to muscle, while T2-weighted images often exhibit a high signal intensity [7]. Nevertheless, these findings lack specificity and fail to yield a definitive diagnosis. To achieve a conclusive diagnosis, histopathological examination, involving the removal of the tumor, and immunohistochemical analysis are imperative [8].

A report on 82 cases of retroperitoneal schwannoma revealed that complete tumor resection was undertaken in 60 cases (73%), while partial resection or biopsy was performed in 22 cases (27%). Despite the rarity of malignant schwannomas, the recommendation is for complete resection in the absence of organ invasion [9]. However, surgical treatment of schwannomas introduces complications, notably postoperative neuropathy. The hypogastric nerve descends from the superior hypogastric plexus, anterior to the abdominal aorta, bifurcating caudally from the common iliac artery, and coursing along the pelvic wall, nearly parallel to the ureter, before reaching the pelvic plexus. Injury to the hypogastric nerve can lead to postoperative dysuria, urinary retention, and defecation issues, necessitating careful management [10]. In our case, although the actual hypogastric nerve was unidentifiable, preservation of the right pelvic plexus resulted in the absence of postoperative voiding dysfunction. A study involving 68 surgically treated cases of sacral schwannoma reported motor dysfunction and numbness in 7 cases (10%), along with decreased peristalsis and dysuria of the intestinal tract in 4 cases (6%). Regarding outcomes, 12 patients (18%) experienced recurrence, with 6 (9%) requiring reoperation [8]. Despite preoperative nerve compression symptoms in our patient, these symptoms improved following tumor resection, and there was no evidence of pain or other neuropathy-induced symptoms or movement disorders.

Various surgical approaches, namely anterior, posterior, and combined, are employed for the resection of sacral neurogenic tumors [[11], [12], [13]]. The choice of approach is determined by factors such as location and size of the tumor, as well as the presence or absence of invasion into adjacent organs. Generally, a posterior approach is recommended for tumors originating below the third sacral nerve (S3). For lesions originating in S1 or S2, a combination of anterior and posterior approaches is suggested [14]. In the current case, our assessment led us to conclude that the tumor likely originated from S1 or L12. This inference was drawn from the proximity of the tumor to the first sacrum on MRI, the absence of evidence indicating sacrum invasion, and the adequacy of an anterior approach for resection. The tumor was resected en bloc, with no residual tumor.

The deep pelvic cavity is anatomically narrow and complex, and the presence of important surrounding structures often complicates en bloc resection of large tumors [[10], [11], [12],^15^]. Additionally, resection of sizable pelvic tumors can lead to substantial intraoperative hemorrhage. Therefore, thorough preoperative evaluation of indications and techniques for resection is essential [15,16]. Intraoperative hemorrhage may occur if the anterior sacral venous plexus is situated on the dorsal surface of the tumor or if tumor-feeding vessels from the internal iliac arteriovenous system are damaged.

Prevention of intraoperative hemorrhage involves various strategies such as balloon occlusion of the abdominal aorta [12], embolization of the internal iliac artery [17], autologous blood transfusion [18], and manipulative hypotensive anesthesia. Despite these measures, reported average intraoperative blood loss ranges from 1600 to 4300 ml, with an average transfusion volume of 1293 mL [11,12].

In this particular case, tumor-feeding vessels were identified, and vascular embolization using Interventional Radiology (IVR) was performed as a hemorrhage prevention procedure before the operation. The first branch of the left internal iliac artery, a feeding vessel for the tumor, was completely occluded, leading to the termination of blood flow to the tumor. Notably, in this instance, the blood loss was limited to 1130 mL, and the surgery was successfully completed without the need for transfusion. This outcome suggests that preoperative IVR-induced embolization of the main feeding vessel is an effective procedure for preventing hemorrhage.

## Conclusion

We report a case of a giant pelvic schwannoma, a relatively rare occurrence. Surgery for large tumors within the pelvic cavity poses a significant risk of massive intraoperative bleeding due to the confined space, wherein vital organs are tightly enclosed, and the proximity of the internal iliac arteriovenous vein and sacral venous plexus. In our case, preemptive embolization of the main feeding vessels was performed using interventional radiology (IVR) before surgery to mitigate the risk of intraoperative hemorrhage. Notably, the procedure was completed without the need for a blood transfusion. Preoperative embolization of the main feeding vessels through IVR emerges as an effective strategy to avert intraoperative hemorrhage during the surgical management of giant pelvic tumors, including schwannomas.

## Patient consent

Written informed consent was obtained from the patient for publication of this case report and accompanying images. A copy of the written consent is available for review by the Editor-in-Chief of this journal on request.

## Ethical approval

A case report is exempt from ethical approval in our institution.

## Author contribution

TK drafted the article. SM revised the manuscript critically. YS assisted with surgery. TN-revised histopathological findings. HO and TF supervised the manuscript preparation. All authors contributed to the study concept and design during submission and approved the final version of the manuscript.

## Registration of research studies

Not applicable.

## Guarantor

Dr. Shoichiro Mukai.

## Provenance and peer review

Not commissioned, externally peer-reviewed.
